# Acacetin, a Natural Flavone with Potential in Improving Liver Disease Based on Its Anti-Inflammation, Anti-Cancer, Anti-Infection and Other Effects

**DOI:** 10.3390/molecules29204872

**Published:** 2024-10-14

**Authors:** Kuihao Chen, Zhe Gao

**Affiliations:** 1 Department of Pharmacology, School of Medicine, Ningbo University, 818 Fenghua Rd., Ningbo 315211, China; 2 Department of Pharmacy, Zhejiang Pharmaceutical University, 666 Siming Rd., Ningbo 315211, China

**Keywords:** liver disease, acacetin, natural flavone, pharmacological activity

## Abstract

Liver disease is a global public problem, and the cost of its therapy is a large financial burden to governments. It is well known that drug therapy plays a critical role in the treatment of liver disease. However, present drugs are far from meeting clinical needs. Lots of efforts have been made to find novel agents to treat liver disease in the past several decades. Acacetin is a dihydroxy and monomethoxy flavone, named 5,7-dihydroxy-4′-methoxyflavone, which can be found in diverse plants. It has been reported that acacetin exhibits multiple pharmacological activities, including anti-cancer, anti-inflammation, anti-virus, anti-obesity, and anti-oxidation. These studies indicate the therapeutic potential of acacetin in liver disease. This review discussed the comprehensive information on the pathogenesis of liver disease (cirrhosis, viral hepatitis, drug-induced liver injury, and hepatocellular carcinoma), then introduced the biological source, structural features, and pharmacological properties of acacetin, and the possible application in preventing liver disease along with the pharmacokinetic and toxicity of acacetin, and future research directions. We systemically summarized the latest research progress on the potential therapeutic effect of acacetin on liver disease and existing problems. Based on the present published information, the natural flavone acacetin is an anticipated candidate agent for the treatment of liver disease.

## 1. Introduction

Liver disease (cirrhosis, viral hepatitis, drug-induced liver injury, and hepatocellular carcinoma) is a global public health problem and has been ranked the 11th leading cause of death all over the world. Approximately 1 out of every 25 deaths is caused by various liver diseases [1]. Due to the changes in living environment and life habits, the number of deaths caused by liver disease is up to 2 million every year [2]. The distribution of liver disease has obvious regional features, for example, cirrhosis is the 9th leading cause of death in Southeast Asia and Europe and the 5th leading cause in the Eastern Mediterranean [3], and the incidence of hepatic fibrosis is very high in developing countries [4]. As early as 2015, the annual healthcare expenditure for Hepatitis was 23.3 USD billion in the United States only [5]. The global burden of liver disease is expected to increase in the future. Despite the rapid development of molecular biological technology and stem cell therapy, we still feel helpless in the treatment of some serious liver diseases. Liver transplantation is the only choice for the therapy of end-stage liver disease, however, less than 10% of global transplantation needs are met at present, and at the same time, serious organ rejection is an important possible complication that may lead to treatment failure [2,6]. In a word, we do not have satisfactory weapons to combat some liver diseases at present. Developing effective drugs is of great significance for the prevention and treatment of liver diseases.

As a center of various substances metabolism, the liver is at a high risk of injury caused by many metabolites. Meanwhile, liver injury or liver dysfunction affects drug metabolism severely [7,8]. It will, in turn, increase the risk of drug usage. Thus, hepatotoxicity must be considered when drugs are developed to prevent various hepatic diseases. Fortunately, numerous studies have suggested that some extracts from fruit and vegetables have strong bioactivities in terms of hepatoprotection, anti-oxidation; more importantly, these extracts exhibit high safety in experiments [9,10,11]. Among these extracts, flavonoids are extremely famous not only because they are abundant in many kinds of fruits and vegetables, but also, they have lots of important pharmacological activities with low intrinsic toxicity. In vitro and in vivo studies have demonstrated the therapeutic effect of flavonoids against many diseases or disorders, such as cardiovascular disease, diabetes, inflammation, cancer, and liver disease [12]. Thus, they gained increasing attention in recent years. Data from PubMed shows that the number of papers about flavonoids is growing at an exponential speed. These studies suggest that flavonoids may serve as candidate agents for multiple disease therapy. Thus far, over 9000 flavonoids have been identified. According to their structures, flavonoids are classified into seven subgroups: flavonols, flavones, isoflavones, anthocyanidins, flavanones, flavanols, and chalcones (Table 1). To date, very few flavonoids are marketed as medicines in clinics, but some flavonoids appear on the market as health care products and skin care products, such as rutin, quercetin, acacetin, and so on. This review summarized the latest progresses of therapeutic efficacy of acacetin, which is a natural flavone extracted mainly from *Robinia pseudoacacia*, on different liver diseases. It might provide a direction to develop a satisfactory drug for the treatment of liver disease.

## 2. Overview of Liver Disease

The liver is a major metabolic organ in humans. It performs numerous physiological processes, such as the detoxification of drugs and metabolites, and the synthesis of proteins and biochemicals essential for digestion, growth, and immunity [20]. The liver works as a factory of the human body. It transforms raw materials from food into small molecules, which are transported to other organs for use.

The liver has so important and complicated functions that it is prone to many diseases. For example, disorders of glucose and lipid metabolism will lead to Non-Alcoholic Fatty Liver Disease (NAFLD, now called metabolic-associated fatty liver disease (MAFLD)), which is the most common chronic liver disease and has a global prevalence of about 25%, ranging from 13% in Africa to 32% in the Middle East [21]. More and more people might suffer from NAFLD as a result of the prevalence of obesity. Patients with NAFLD often have one or more components of the metabolic syndrome (MS), like dyslipidemia, insulin resistance, and hypertension. Though most patients with NAFLD do not have symptoms, NAFLD could develop to liver fibrosis/cirrhosis and subsequently to liver cancer [22]. However, there are no satisfactory therapeutic strategies against NAFLD in the clinic. Weight loss and dietary modification remain the main choices before a specific drug is approved to improve NAFLD [22,23].

Hepatocellular carcinoma (HCC) is the 5th most common cancer and the 4th leading cause of cancer-related deaths worldwide. HCC accounts for over 80% of primary liver cancer. It usually develops within a background of advanced chronic liver disease, mainly related to hepatitis B virus (HBV) and hepatitis C virus (HCV). However, NAFLD is rapidly becoming a major cause of HCC, as the prevalence of obesity and diabetes increases [24,25]. Currently, multiple approaches, including surgery, chemical drugs, radiation, and targeted immunotherapy, are applied for HCC therapy. Surgical resection is the main therapeutic strategy for HCC at an early stage, but usually, HCC is diagnosed at an advanced stage. In this instance, chemotherapy, immunotherapy, and nanotechnology-based drug therapy approaches are relatively better choices [25,26,27]. Additionally, natural compounds from plants seem to have great potential in HCC therapy, and they might be new choices [28,29,30,31].

The virus is another big threat to the liver. Multiple viruses could induce a liver disease called viral hepatitis, including hepatitis A, hepatitis B, hepatitis C, hepatitis D, and hepatitis E. Viral hepatitis is also a severe global public health problem. It is estimated that approximately 57% of cirrhosis and 78% of hepatocellular carcinoma are associated with hepatitis B or hepatitis C. Viral hepatitis accounts for approximately 1 million deaths each year [2,32,33]. Except for hepatitis virus B, which is a DNA virus, other hepatitis viruses are all single-stranded RNA viruses. Worldwide, approximately 1.4 million new cases of hepatitis A are reported each year, 360 million people are living with hepatitis B, 71 million people are living with hepatitis C, there are 12 million hepatitis D virus infections, and 20 million people are suffering from hepatitis E virus [34]. The diagnosis of viral hepatitis depends on serological assays. Fortunately, acute hepatitis A is a self-limited disease. Most patients with hepatitis A can recover spontaneously in 2 months without anti-viral treatment. Therefore, the therapeutic strategy for hepatitis A is supportive care. Less than 5% of hepatitis A cases advance to liver failure. However, liver-protective treatment is needed when there are severe symptoms. No specific drug was developed to treat acute hepatitis A at present [35]. In 2019, the global prevalence of chronic hepatitis B was approximately 3.5%. Hepatitis B is the leading cause of cirrhosis and HCC. The pathogenesis of hepatitis B is very complicated. It is mainly the adaptive immune response that mediates liver injury in patients infected with hepatitis B virus (HBV). The integration of HBV into the host genome leads to the persistent expression of hepatitis B surface antigen (HBsAg), which induces an immune response [36,37]. This is why the treatment of hepatitis B is quite difficult. The ultimate goal of hepatitis B therapy is to prevent the occurrence of cirrhosis, HCC, and liver failure. Anti-viral therapy was demonstrated to be beneficial for the prevention of liver fibrogenesis and hepatocarcinogenesis [38,39]. Pegylated interferon alpha and nucleotide analogs are common anti-viral agents currently. Considering the importance of immune response in hepatitis B, small natural compounds extracted from plants that have the potential for anti-virus and immune regulation might serve as candidate agents for treatment of hepatitis B. Hepatitis C is caused by the hepatitis C virus (HCV) infection. HCV could evade the immune system and cause chronic hepatitis, which often advances to fibrosis and cirrhosis [40]. It has been reported that an HCV co-infection with HBV would have an increased risk for cirrhosis and HCC [41]. In many countries, chronic HCV infection is the major cause of having a liver transplant. Unlike hepatitis A and hepatitis B, no effective vaccine can be used to prevent hepatitis C. HCV could be eliminated by direct-acting anti-viral agents (DAA), such as sofosbuvir, simeprevir, and so on, and the sustained virologic response (SVR) rates have reached 95–99% [42,43]. Besides DAA, developing host-targeting agents (HTA), which can target hepatocyte molecules required for the HCV life cycle, will be an effective therapeutic strategy for hepatitis C. Several HTAs have shown therapeutic effects in animal models and are being evaluated in phase 2 and 3 clinical trials [44,45]. Chronic hepatitis D is the most severe type of viral hepatitis. Approximately 5% of people infected with HBV are co-infected with hepatitis D virus (HDV). It was found that the propagation of HDV in hepatocytes needs the help of HBV [46]. The pathogenesis of HDV infection is not very clear until now. Except for innate and adaptive immune responses, which may take part in mediating liver damage, the inflammatory response induced by HDV antigens, including endoplasmic reticulum (ER) stress and necroinflammation, might promote the development of cirrhosis and HCC [47,48]. Among all viral hepatitis, hepatitis D is the most difficult to treat. In the future, agents targeting the entry of virus into hepatocytes, preventing virus prenylation and assembly, and strengthening the anti-HDV immune system will be beneficial for hepatitis D therapy. Hepatitis E is the 5th known form of viral hepatitis. Like HAV, the hepatitis E virus (HEV) mainly causes acute hepatitis, and the majority of acute HEV infections do not need any special therapy in immunocompetent patients. However, immunocompromised patients who are infected with HEV may advance to chronic hepatitis and cirrhosis because HEV cannot be eliminated by the patients’ immune system [49,50,51]. Pegylated interferon-alpha and ribavirin showed therapeutic effects on chronic HEV infection. However, some side effects, including skin reactions, can be caused by ribavirin. An in vitro study showed that the natural compound silvestrol could suppress HEV replication. The virus can be cleared in mice treated with silvestrol, though it has not been tested in humans [49,52].

The liver is sensitive to injury due to its role in the metabolism and detoxification of a vast majority of drugs. The metabolites of drugs can cause unexpected damage to hepatocytes. Drug-induced liver injury (DILI) is a common clinical problem and is the most frequent cause of acute liver failure (ALF) [53,54]. In the United States, about one-half of ALF cases were caused by acetaminophen (APAP) overdose [55]. In fact, DILI is one of the most important reasons for stopping drug development, withdrawal, and post-marketing regulatory actions [56,57]. The mechanism of hepatocyte death caused by DILI is mainly due to organelle stress, such as ER stress and mitochondrial stress, which leads to necrosis or apoptosis [58,59]. The therapeutic strategy of DILI is to clear poisons, suppress hepatocyte death, and prevent liver failure.

Hepatic fibrosis is a histopathological change in the liver. Many liver diseases, including hepatitis B, hepatitis C, chronic liver injury, and NAFLD, cause sustained hepatocellular damage leading to fibrosis, which is the early stage of cirrhosis. Globally, liver cirrhosis is currently the 11th most common cause of death [2]. Hepatic fibrosis is an indicator of the decompensation of chronic liver disease and has a significant impact on patient mortality. The development of fibrosis is complicated, and it is a result of multiple factors working together. The most studied cellular molecules and events involved in the development of hepatic fibrosis include PDGF signaling, TGF-β signaling, oxidative stress, and so on [60]. It has been found that hepatic fibrosis is reversible after the underlying etiological agent is removed. However, the reversal often occurs too slowly, especially in advanced fibrosis. Thus, some medicines are needed to prevent fibrosis development. Although there are many anti-fibrotic candidate agents showing significant effects in animal models, their anti-fibrotic effects are not as well as expected in clinical trials [60,61,62,63].

## 3. Overview of Acacetin

### 3.1. Source and Structure of Acacetin

Acacetin is one of the natural flavonoids that can be found in plants and fruit. In 1970, an acacetin derivative, acacetin-7-glucurono-(1 lead to 2)-glucuronide, was first discovered as a glycoside in the leaves of clerodendron trichotomum [64]. Then, an acacetin monomer was identified in the leaves of the thistle [65], and acacetin can also be found as a metabolite of some flavonoids in vivo [66]. Now, we know that acacetin is present in the roots, leaves, flowers, and aerial part of as many as 90 plants, including the leaves of *Tueradiffusa*, the leaves of *Propolis*, the whole plant of *Agastache rugosa*, the whole plant of *Moslascabra*, the aerial part of *Artemisia afra*, and so on [67].

**Figure 1:**
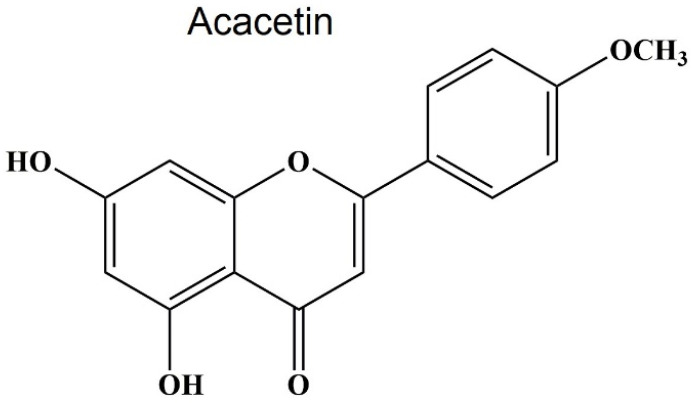
Structure of acacetin. The molecular formula and weight of acacetin are C_16_H_12_O_5_ and 284.26 g/mol.

Structurally, acacetin belongs to flavones, which is the most important type of flavonoids. Flavones that include luteolin, apigenin, acacetin, wogonin, baicalain, and tangeretin are abundant in green tea, red pepper, broccoli, olive oil, oregano, and citrus fruits [68,69]. The basic chemical structure of flavones is C6-C3-C6, and it contains a ketone group at C4. They are composed of the flavone core called 2-phenyl-1,4-benzopyrone. The largest differences between flavones and other flavonoids are that flavones have a double bond between C2 and C3 in the flavonoid skeleton and no substitution at the C3 position [14,70]. Acacetin is also called 5,7-dihydroxy-4′-methoxyflavone (Figure 1).

The molecular formula of acacetin is C_16_H_12_O_5_ and its molecular weight is 284.26 g/mol [71]. Besides being extracted from plants, acacetin can also be synthesized artificially with different approaches, but the most efficient method was reported by Zhao Y. et al., where 4-Dimethylaminopyridine (DMAP) acted as a catalyst for the two-component condensation between phenols and β-ketoesters [67,72]. Like other flavones, natural acacetin mainly exists in the form of glycosides, including tilianin, acacetin 7-(2′-acetylglucoside), acacetin 7-O-(6-O-malonylglucoside), which exist in dairy foods and can be absorbed quickly [73]. However, the acacetin monomer is extremely different. Due to the weak solubility (≤119 ng/mL) and low stability in an acid environment, the oral bioavailability of acacetin is as low as 2.34%. At the same time, acacetin can be metabolized in various tissues with a clearance speed of 199 ± 36 mL/min/kg [74]. As many as 31 metabolites of acacetin were detected in rats, including diosmetin, luteolin, apigenin, and naringenin [75]. These metabolites have similar biological activities as acacetin. All these features (weak solubility, low stability, and bioavailability) of acacetin prevent its successful application in clinics. Given the multiple pharmacological activities of acacetin, it is worth studying how to decorate the structure of acacetin or create nanostructured carriers to improve bioavailability. Few studies were performed to change the structure of flavones at the level of C rings. Most investigations focused on changing the hydroxyl or methoxy group because of their critical roles. For example, a water-soluble acacetin prodrug called phosphate sodium salt of acacetin was synthesized. This prodrug can be successfully converted to acacetin in the blood. Importantly, it dramatically improved the concentration and duration of acacetin in blood to meet the therapeutic need [76].

### 3.2. Pharmacological Activities of Acacetin

The phenolic hydroxyl groups make acacetin strong anti-oxidants against reactive oxygen species (ROS) that participate in the initiation of many pathophysiological processes, such as steatohepatitis, hepatic fibrosis, acute liver injury, and liver cancer. It is well known that mechanisms of anti-oxidant action include: one, suppressing the activities of enzymes that are related to ROS production, two, scavenging ROS directly, three, increasing production of anti-oxidants [77]. Acacetin, the main flavone compound extracted from the shell of *Couepiabracteosa*, exhibited a strong scavenging capacity against ROS and reactive nitrogen species (RNS) directly. The shell (mainly acacetin) scavenges H_2_O_2_, HOCl, ^•^NO, and ONOO^−^ with the IC_50_ of 894 ± 3 μg/mL, 25.3 ± 0.4 μg/mL, 485 ± 2 μg/mL, and 53 ± 1 μg/mL, respectively [78]. Acacetin (80 mg/kg, 2 days) can suppress the expression of enzymes that promote the production of ROS, including inducible nitric synthase (iNOS), cyclooxygenase-2 (COX-2) in sepsis-induced acute lung injury animal models [79]. Other studies have shown that acacetin (10 mg/kg, 7 days; 10 mg/kg, 16 weeks) also can upregulate the expression of anti-oxidants, including superoxide dismutases (SODs), heme oxygenase 1 (HO-1) in acute lung injury, cardiac hypertrophy, diabetes-induced cardiomyopathy, and myocardial ischemia/reperfusion injury animal models [80,81,82,83,84]. Mechanistically, sirt1/AMPK and Nrf-2/HO-1 signaling pathways are proved to be involved in the process. Of note, acacetin can also promote ROS production in some carcinoma cells, such as gastric carcinoma cells, breast cancer cells, leukemia cells, and human osteosarcoma cells [85,86,87,88]. However, the authors did not explain the mechanism of ROS production induced by acacetin. The contrary effect of acacetin on ROS in different cells is an interesting topic. At least, it indicates that acacetin could target multiple cellular molecules or events. Further studies are needed to figure out the underlying mechanism of acacetin-promoting ROS production.

Tumorigenesis is a very complicated process. The suppressive effects of flavones on cancers have been reported, and multiple cellular events and signaling pathways are involved in this process. Inhibition of carcinoma cell proliferation is the basic capacity of most anti-cancer drugs. It was found that acacetin exhibited the potential for anti-proliferation and apoptosis-induction in many cancer cell lines. Such as HCC cell lines [89,90], breast carcinoma cells [86,91], lung cancer cells [92,93], prostate carcinoma cells [94,95,96], colon carcinoma cells [97,98], leukemia cells [87,99,100], gastric carcinoma cells [85,101,102]. The mechanism of acacetin inhibiting carcinoma cell proliferation is a little bit different among cancers. In breast cancer cells, cell apoptosis induced by acacetin was due to the loss of mitochondrial membrane potential and release of cytochrome C and activation of the stress-induced protein kinase/c-Jun NH4-terminal kinase 1/2 (SAPK/JNK1/2) signaling pathway, but ERK1/2 is not activated in MCF-7 cells [91]. However, acacetin can induce ERK1/2 activation in other kinds of breast cancer cells, such as T-47D and MDA-MB-231 [86]. In prostate carcinoma cells, acacetin inhibited STAT3 and the AKT/NF-κB signaling pathway and made the cells stay in G1 and/or G2-M stages via increasing Cip1/p21 and decreasing CDK2, CDK4, and CDK6. In vivo studies showed that acacetin (50 mg/kg, 30 days; 50 mg/kg, 48 days) significantly suppressed tumor growth, including a decrease in tumor volume and weight [94,95,96]. Upregulation of Bax, loss of mitochondrial membrane potential, downregulation of Bcl-2, and suppression of the AKT-mTOR pathway were responsible for acacetin-induced leukemia cell apoptosis. In the colon carcinoma cells, acacetin-induced cell death was mediated by ROS. Interestingly, promoting ROS production is a common mechanism of acacetin-induced cancer cell apoptosis. Until now, it has not been explained why acacetin could promote ROS production in cancer cells while suppressing that in normal cells suffered from dangers.

Inflammation takes part in the advancement of many diseases, such as every phase of NAFLD, acute liver injury, and liver cancer. The anti-inflammatory effect of acacetin may be partially related to its suppression of ROS induced by various stimulators. Acacetin (50 mg/kg, 8 weeks; 10 mg/kg, 12 weeks; 150 mg/kg, 9 days) decreases gene or protein levels of tumor necrosis factor-α (TNF-α), interleukin-6 (IL-6), and/or interleukin-1β (IL-1β) in type 2 diabetic rats [103], obese mice [104], mice with colitis [105], LPS-stimulated human periodontal ligament (PDL) cells [106], LPS-induced neuroinflammation mouse model [107], and LPS-induced acute lung injury in mice [82]. TNF-α, IL-6, and IL-1β are well-known markers of inflammation. Further studies showed that the Nrf2 pathway, AMPK pathway, PI3K/AKT/IKK, and MAPK participated in acacetin-induced decreases in TNF-α, IL-6, and IL-1β [108,109]. In addition, acacetin (10 mg/kg, 8 weeks) alleviates inflammatory response in obese mice through modulating Treg/Th17 balance via targeting MiR-23b-3p [110]. NLRP3 inflammasome or NLRP3 signal pathway plays an important role in inflammation. Acacetin also inhibits the expression of NLRP3 in cerebral ischemia-reperfusion injury [111], suppresses NLRP3 expression and enhances gp78-mediated NLRP3 ubiquitination in depression-associated dry eye disease [112], and reduces the expression of NLRP3 in mice with Alzheimer’s disease [113]. These studies indicate that acacetin exhibits strong inhibitory effects against inflammation in multiple animal models via different mechanisms. However, the dominant mechanism is still unknown.

The effect of acacetin on combating viruses and bacteria was also reported. As early as 1994, it has been reported that acacetin-7-O-beta-D-galactopyranoside extracted from Chrysanthemum morifolium showed anti-HIV activity with relatively low toxicity [114]. Acacetin could inhibit HIV expression in TNF-α-treated OM-10.1 cultures. The inhibitory effect of acacetin on HIV activation was associated with interfering the viral transcription [115]. However, the exact mechanism was unknown. As the prevalence of SARS-CoV-2 at the beginning of 2020, it was found that acacetin, the main active ingredient of JingFangBaiDu San, could bind to angiotensin converting enzyme II (ACE II) and spike protein, which inhibits virus entry and replication in host cells [116]. Acacetin inhibited HBsAg and HBeAg synthesis by 43.3% and 41.2%, respectively, due to its interaction with Asp205 of wild-type HBV polymerase and Tyr203-Asp205 of mutant HBV polymerase [117]. Acacetin exhibited great inhibitory activity against wild-type H1N1 and H3N2 viruses. Moreover, acacetin also inhibited drug-resistant viral strains, though the exact mechanism was still unknown [118,119]. In addition, acacetin exhibited inhibitory effects against both gram-positive and gram-negative bacteria [120]. Listeriolysin O (LLO) is the major virulence factor of *Listeria monocytogenes*. Acacetin can inhibit LLO activity and promote the clearance of *Listeria monocytogenes* by directly binding to the second membrane-inserting helix bundle of LLO domain 3 [121]. Acacetin was demonstrated to be an effective inhibitor of suilysin, which is the most important virulence factor of streptococcus suis. Furthermore, it alleviated streptococcus suis mediated cell injury by inhibiting the inflammatory response. However, it cannot suppress bacterial growth [122]. However, like commonly used anti-biotic drugs, acacetin can target bacterial cell wall biosynthesis, bacterial protein synthesis, and bacterial DNA replication and repair of *Escherichia coli* O157:H7 [123]. It was reported that the inhibitory effects of acacetin against *Staphylococcus aureus*, *Enterococcus faecalis*, *Escherichia coli*, and *Pseudomonas aeruginosa* had minimum inhibitory concentration (MIC) values of 0.16, 0.32, 0.32, and 0.35 mg/mL [124]. Further study showed that acacetin inhibited the activity of sortase A, an important virulence factor for bacterial pathogenesis, by binding with residues Arg-139 and Lys-140 at the pocket. Acacetin was able to significantly increase the survival rate of animal model infected with *Staphylococcus aureus* [125]. Additionally, acacetin effectively improved mouse model of streptococcus pneumoniae infection by inhibiting the pore-forming activity of pneumolysin (PLY), which is the major virulence factor of streptococcus pneumoniae [126]. According to the present studies, the main mechanism of anti-bacterial activity of acacetin is to inhibit the activity of virulence factors. This effect of acacetin is quite different from traditional anti-biotic drugs. It provides a novel strategy for the development of anti-biotic drugs.

Both type 2 diabetes and NAFLD belong to metabolic diseases. Researchers found that type 2 diabetes has a two-fold increased risk of NAFLD, and vice versa [127]. The pathogenesis of type 2 diabetes is still not fully understood, though numerous studies have been conducted in the past years. At present, oxidative stress, inflammatory cytokines, inflammatory signaling molecules, and ER stress have been demonstrated to play important roles in the advancement of type 2 diabetes [128,129]. Studies showed that diabetes or diabetes-related diseases could be alleviated by acacetin in animal models. Many signaling molecules or cellular events are potential targets of acacetin. For example, it was reported that acacetin reduced blood glucose levels in streptozotocin (STZ)-hyperglycemic mice via eliminating oxygen free radicals [130]. In high-fat diets combined with STZ rats, oral administration of acacetin (50 mg/kg, 8 weeks) significantly lowered blood glucose and improved hepatorenal dysfunction. Since the expression levels of TNF-α, IL-6, and IL-8 were reversed by acacetin in diabetic rats, the potential mechanism of acacetin-mediated decrease of blood glucose might be associated with its anti-oxidant and anti-inflammatory activities [103]. In muscle and HepG2 cells, acacetin could promote glucose uptake by enhancing GLUT4 translocation to the plasma membrane and activating the CaMKII-AMPK pathway [131]. Acacetin also exhibits a protective effect on diabetes-induced endothelial cell injury [132], cardiomyopathy [83], and kidney disease [133] by targeting PPARα, the Sirt1/Sirt3 signaling pathway, and dipeptidyl peptidase-4 (DPP4). Hence, acacetin may be a promising candidate agent for diabetes treatment.

Although so many pharmacological activities of acacetin have been confirmed, it seems that most of these activities of acacetin are associated with the anti-oxidant and anti-inflammatory effects. Inflammatory reactions and oxidative stress are the basic events and play important roles in the advancement of liver diseases. Considering the pharmacological activities of acacetin, the therapeutic effects of acacetin on liver diseases are worth expecting.

## 4. Therapeutic Effects of Acacetin on Liver Diseases

### 4.1. Anti-Hepatocellular Carcinoma

As mentioned above, HCC has a high morbidity and mortality all over the world. Surgery is accepted as the first choice for most HCC cases, but medication is still an indispensable treatment mode. All HCC cases will receive drug therapy after surgery. However, not an ideal anti-HCC drug can be chosen in the clinic at present. Fortunately, natural flavonoids, such as acacetin, exhibited great potential in anti-HCC in vitro studies. The first study of acacetin on HCC was performed in 2004. Acacetin induced HepG2 cell apoptosis and blocked cell cycle progression in the G1 phase via upregulating p53 and Fas/Fas ligand apoptotic system [84]. In addition, STAT3 has been proven to play an important role in regulating proliferative, survival, metastasis, and angiogenesis genes in HCC, and STAT3 activation could accelerate HCC progression [134]. Thus, STAT3 may be a therapeutic target of HCC [135,136,137]. Acacetin was found to suppress Huh-7 and HepG2 cells by inhibiting both STAT3 activation and the upstream kinases c-Src, JNK1, and JNK2 [89]. The anti-tumor effect of acacetin in vivo was studied in BALB/c mice subcutaneously transplanted with HepG2/RARγ liver cancer cells. After 3-week treatment, acacetin significantly decreased tumor volume by 61.1% with the dose of 30 mg/kg compared with vehicle control due to its induction of cancer cell apoptosis through antagonizing the non-genomic effect of RARγ on AKT and p53 [138]. This is an important advancement in studying the pharmacological effects of acacetin, although the therapeutic effect of acacetin on HCC should be further confirmed in more animal models.

Many studies have suggested that the tumor microenvironment is required for supporting the development of cancers, including HCC. Recently, it has been found that tumor-associated macrophages (TAMs) are believed to occupy an irreplaceable position in the development of HCC, including immunosuppression, immune escape, angiogenesis, tumor invasion, and metastasis [139]. Thus, regulating TAMs may be another important therapeutic strategy for HCC. It was reported that luteolin, a natural flavone, inhibited the migration of Lewis lung carcinoma cells by suppressing TAM-secreted CCL2 [140]. Another natural flavone tricin was also found to modulate tumor microenvironment in colon cancer mouse model [141]. However, the effect of acacetin, which has a similar structure to luteolin and tricin, on modulating tumor microenvironment, or TAMs, has not been reported. This is a research direction worth exploring. Meanwhile, considering the roles of inflammation and oxidative stress in HCC progressing, we believe that the potential of acacetin in improving HCC is expectable. Meanwhile, more efforts should be made to uncover the underlying mechanism in the future.

### 4.2. Anti-NAFLD

NAFLD is the leading chronic liver disease worldwide and is characterized by the accumulation of more than 5% of fat in liver cells [142]. NAFLD summarizes a broad disease spectrum ranging from simple steatosis to non-alcoholic steatohepatitis (NASH). NASH patients are at high risk of progressing to hepatic fibrosis, cirrhosis, and HCC. The imbalance of energy metabolism is responsible for the development of NAFLD. Energy entering the liver excesses the liver’s ability to dissolve or export it as lipoproteins, leading to a net accumulation of energy in the liver as triglycerides. The excessive triglycerides synthesized in the liver could be mainly stored in two kinds of cells. One is adipocyte, another is hepatocyte. This is why the prevalence of NAFLD is usually linked to obesity and type 2 diabetes [143,144]. One of the therapeutic strategies of NAFLD is focusing on modulating the energy metabolism in the liver, such as suppressing lipogenesis and promoting lipolysis and fatty acid β-oxidation. Additionally, inflammatory and/or immune-related events play as independent stimulators in NAFLD pathogenesis, such as inflammasome activation, altered innate immune signaling, macrophage responses, and so on. Thus, modulating inflammation and/or immune is believed to be beneficial for NAFLD therapy.

In 2017, acacetin was first demonstrated to inhibit adipogenesis in 3T3-L1 adipocytes and in HFD-induced obese mice. The inhibitory effect of acacetin on adipogenesis is due to the suppression of CCAA/enhancer-binding protein and reduction in the inflammatory mediators [145]. Five years later, the results from the same group show that acacetin protects against NAFLD by suppressing lipid accumulation and modulating the inflammatory response. Not only can acacetin inhibit the gene expressions of transcription factors related to lipogenesis, but it can also promote lipolysis and fatty acid β-oxidation in the liver [104]. Our group further revealed the mechanism of acacetin for improving NAFLD. We found that acacetin (20 mg/kg, 16 weeks) inhibited lipid accumulation by suppressing ER stress and ferroptosis. Meanwhile, ER stress works as the upstream signal of ferroptosis in lipid accumulation [146]. It also has been reported that acacetin (20 mg/kg, 14 days) improved insulin resistance, body weight, and energy metabolism disorder via regulating Treg/Th17 balance and promoting white fat browning in obese mice [110,147]. Although the lipid situation in the liver was not detected in these two studies, the regulating role of acacetin in energy metabolism might be beneficial to improve NAFLD. We have to admit that the study of acacetin on protecting NAFLD is still in its infancy, there is lots of work to do in the future. Such as the mechanisms of action and the targets of acacetin are not fully understood.

### 4.3. Anti-Fibrosis

Like other organ fibrosis, hepatic fibrosis is also characterized by the accumulation of extracellular matrix (ECM). Hepatocyte death caused by toxic viruses and metabolites is the initial event of fibrosis. Then, compounds released from dead hepatocytes activate hepatic stellate cells (HSCs) and Kupffer cells. HSCs are responsible for producing ECM components, including types I, III, and IV collagens, fibronectin, and laminin. While activated Kupffer cells can provide a pro-fibrogenic environment and maintain activation of HSCs [60,148]. Hepatic fibrosis is reversible, and it is an intermediate stage of chronic liver diseases, such as NAFLD and viral hepatitis, progressing to cirrhosis. Due to the reversibility of fibrosis, removal of the underlying etiological cause of injury is the most important step in the treatment of hepatic fibrosis. Other therapeutic strategies include suppression of hepatocyte apoptosis, inhibiting HSC activation, and hepatic inflammation. In animal models, many specific inhibitors displayed therapeutic efficiency in fibrosis, such as cannabinoid receptor 1 (CB1) inhibitors, IL-13 antibodies, TIMP1 antibodies, and hedgehog inhibitors. However, no an anti-fibrotic drug has been approved by Food and Drug Administration (FDA) currently. Fortunately, many studies have found the anti-fibrotic effect of acacetin in animal models. Acacetin (20 mg/kg, 6 weeks; 50 mg/kg, 6 weeks) inhibits the hypertension-associated cardiac fibrosis and aortic fibrosis via regulating TGF-β/Smad3, AKT/mTOR, estrogen signaling pathways and reducing the release of inflammatory factors [149,150]. In rats performed with abdominal aorta constriction (AAC), acacetin ameliorates myocardial fibrosis by Sirt1-mediated activation of AMPK/PGC-1α signal molecules [81]. Additionally, network pharmacology and transcriptomic analysis indicate that acacetin might inhibit liver fibrosis by regulating Jak-STAT and PI3K-Akt-FoxO signaling pathways [151]. To date, no experiment data was published to reveal the effect of acacetin against hepatic fibrosis. However, based on the pharmacological activities of acacetin in anti-oxidation and anti-inflammation and the inhibitory impact of acacetin on fibrosis in the cardiovascular system, along with network pharmacology prediction and the anti-hepatic fibrosis properties of other flavones, such as apigenin and luteolin [152,153,154], we could make an assumption that acacetin may have the potential of reversing hepatic fibrosis. Thus, without doubt, evaluating the therapeutic effect of acacetin on hepatic fibrosis is an attractive research direction.

### 4.4. Anti-Acute Liver Injury

Acute liver injury (ALI) is also a common disease characterized by the massive death of hepatocytes within a short period of time. It is a big threat to the health of patients as the dramatic decline in their liver function [155]. The etiology of ALI includes drug-induced liver injury, ischemia-reperfusion liver injury, autoimmune liver injury, and viral liver injury [156,157]. Viral is the leading cause of ALI in developing countries, while drugs are the main cause of ALI in Western countries [158]. The compounds released from dead hepatocytes trigger a cascade of immune/inflammatory responses, which play a critical role in the progression of ALI to acute liver failure [159]. Treatment options for ALI are limited at present, therefore it is largely dependent on supportive care. Liver transplantation is the final choice for all decompensated liver diseases. Considering the importance of immune responses in ALI pathogenesis, it may provide a novel therapeutic strategy for improving ALI. A study has shown that intraperitoneal injection of acacetin (50 mg/kg, 6 h) inhibits d-galactosamine (GalN) and lipopolysaccharide (LPS)-induced liver injury via suppressing TLR4 signaling and inflammatory response [154]. Recently, we found that acacetin (20 mg/kg, 4 h) could protect acetaminophen (APAP)-induced liver injury in mice by inhibiting ER stress. Moreover, molecular docking revealed the direct interaction of acacetin with peroxisome-proliferator-activated receptor gamma (PPARγ) [160,161]. However, how PPARγ regulates ER stress needs further study. Of interest, acacetin alleviates liver injury caused by renal ischemia-reperfusion via reducing inflammatory response in a dose-dependent manner in mice [162]. All these studies indicate the potential for acacetin in the treatment of ALI.

## 5. Pharmacokinetics and Toxicity Studies

Although flavones have various pharmacological properties, their bioavailability is very low. The maximum plasma concentration of acacetin was 1334.9 ng/mL after administration of acacetin intravenously (5 mg/kg). Additionally, it was found that the plasma concentration of acacetin after intravenous administration declined rapidly with a half-life of 1.48 h [163]. In most animal experiments, acacetin was administered by gavage. However, interestingly, it was reported that only metabolites but not parent compounds could be detected after oral administration of acacetin with 50 mg/kg in rats [164]. There are as many as 31 metabolites of acacetin that can be detected in rats. Most of the metabolites of acacetin were generated by phase II metabolism. The pathway for acacetin metabolism includes the loss of CH_2_, hydrolysis, oxidation, methylation, N-acetylation, sulfate conjugation, and glucuronide conjugation [75]. In another study, acacetin could be detected, and the maximum plasma concentration was 31.92 ng/mL when oral administration of *Buddlejalindleyana* Fort. Extract, which contained seven active components, linarin, rutin, luteolin, quercetin, apigenin, acacetin, and acteoside [165]. Liu H. et al. synthesized an acacetin prodrug. The maximum plasma concentration of acacetin was as high as 2483 ng/mL in beagle dogs administrated with the acacetin prodrug (6 mg/kg) by intravenous, and the half-life of acacetin is approximately 1.5 h [76]. However, the plasma concentration of the acacetin prodrug administered by gavage is unknown. Anyway, we have moved a big step forward in clinical application of acacetin by structural modification.

As a natural product, the most important advantages of acacetin may be low toxicity, tolerability, and fewer adverse effects. Although a study of acacetin on cytochrome P450 showed the inhibitory effects of acacetin on different isoforms of cytochrome P450, especially CYP1A2, CYP2B1, CYP2C11, CYP2D1, CYP2E1, and CYP3A2, which indicates the high risk of acacetin in toxicity and drug interaction [166]. It has been reported that intraperitoneal administration of acacetin (50 mg/kg) did not induce any harmful impact on male reproductive and urinary systems in mice treated for 10 days continuously [167]. Additionally, no obvious pathological changes in the liver, lung, and kidney of mice were observed when acacetin (50 mg/kg) was administered by intraperitoneal injection every 2 days for 3 weeks [101]. The acute toxicity of acacetin has not been reported yet. However, the acute toxicity of the acacetin prodrug, which can be completely converted to acacetin in plasma, was reported, and the LD_50_ value was 721.7 mg/kg (equal to 502.5 mg/kg of acacetin) when administered intravenously in mice [76]. According to the present studies, the direct toxicity of acacetin is very low at the dose of no more than 50 mg/kg, which is the maximal concentration commonly used in multiple animal models with different diseases.

## 6. Discussion and Conclusions

### 6.1. Current Problems

Current studies show that acacetin exhibits attractive prospects for treatment of various disorders, such as cancer, liver diseases, obesity, diabetes, and lung injury. There are still some obstacles on the road to clinical application of acacetin. The studies on pharmacological activities of flavones have sustained for many decades. However, the poor solubility of flavones, including acacetin, limited their clinical use seriously. The good news is that a water-soluble acacetin prodrug has been synthesized, and its therapeutic effects on cardiovascular diseases have been demonstrated when it is administered intravenously. However, the bioavailability of the acacetin prodrug via oral administration is undetermined. In order to improve the absorption of oral administration of acacetin, novel drug delivery approaches, or structural modifications should be taken into consideration. Several reports reveal that the metabolism of acacetin in plasma is very fast. The half-life of acacetin in plasma was found to be approximately 1.5 h, whenever it was administrated as an acacetin itself or acacetin prodrug intravenously. Thus, future research should also focus on how to prolong the half-life of acacetin. In the past 5 years, the number of publications about acacetin has increased rapidly. Numerous studies were only performed in an in vitro system. There are no ideal animal models, which could completely mimic the natural development of the related diseases, to evaluate the pharmacological effects of acacetin. To better evaluate the efficacy of acacetin, big animal models are needed, such as dogs, pigs, and monkeys. The pharmacological activities of acacetin, including anti-oxidation, anti-tumor, anti-inflammation, and anti-obesity, have been demonstrated in vivo or in vitro studies these years. However, the exact mechanisms are still unclear. Therefore, network pharmacology and molecular docking should be employed to further reveal the targets and mechanisms of action of acacetin in the future. Meanwhile, the systematic study on the pharmacokinetics and toxicity of acacetin has not been reported, either. Thus, in summary, there is lots of work to do before acacetin will be brought to clinical trial.

### 6.2. Conclusions

**Figure 2:**
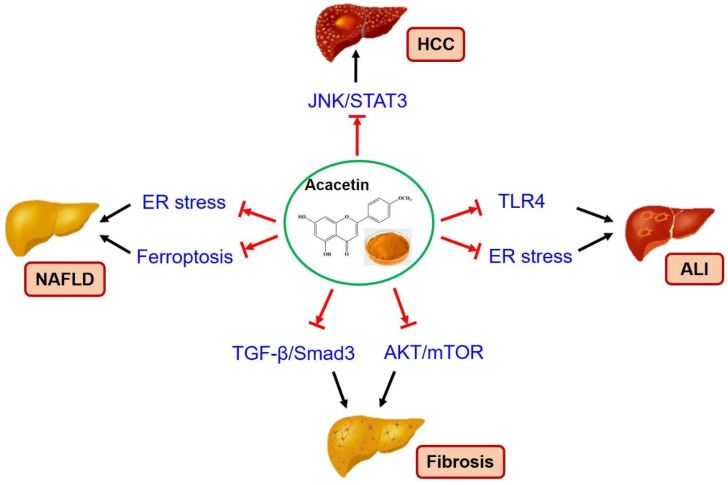
Possible mechanisms involved in the therapeutic effect of acacetin on liver disease. JNK/STAT3 signaling pathway is activated in HCC; TLR4 signaling, and ER stress promote ALI development; ferroptosis/ER stress participates in the pathogenesis of NAFLD; TGF-β/Smad3 and AKT/mTOR signaling pathways play a critical role in fibrosis. Acacetin improves liver disease by inhibiting signaling pathways above in vivo and/or in vitro. HCC: hepatocellular carcinoma, NAFLD: Non-Alcoholic Fatty Liver Disease, ALI: acute liver injury [168].

The multiple pharmacological benefits of acacetin on various disorders caught worldwide attention. However, the studies of acacetin on liver disease therapy are just beginning. As of the end of 2023, there were only 47 publications in PubMed when searched with the key words “acacetin liver”. Here, we summarized the pharmacological activities of acacetin and the present studies on the potential therapeutic effect of acacetin on liver diseases, including HCC, NAFLD, liver fibrosis, and ALI. Acacetin displayed anti-HCC activity via suppressing cell proliferation or inducing cancer cell apoptosis related to the inhibition of JNK/STAT3, AKT, and p53. Acacetin improved NAFLD by promoting lipolysis and fatty acid β-oxidation, modulating inflammation, suppressing ER stress, and ferroptosis. Acacetin even could promote white fat browning, leading to the improvement of NAFLD. In addition, acacetin protected ALI induced by LPS, APAP, or renal ischemia-reperfusion via suppressing TLR4 signaling and inflammatory response and ER stress. Though, the therapeutic effect of acacetin on liver fibrosis has not been reported. It is well known that the fibrosis-related molecules, including TGF-β/Smad3, AKT/mTOR, AMPK/PGC-1α, and inflammatory factors, could be regulated by acacetin (Figure 2). The protective effect of acacetin on liver fibrosis is discussed and predicted here, but it needs to be confirmed in the future. To date, acacetin has been reported to modulate the activation of various signaling molecules and intracellular events, such as JNK/STAT3, AKT/mTOR, TGF-β/Smad3, MAPK, COX-2, Nrf-2, ER stress, and ferroptosis. These are believed to be the basis for acacetin preventing liver diseases. In addition, there is still not enough data available on the pharmacokinetics and toxicity of acacetin.

In this review, we focused on the advances of acacetin in preventing liver diseases and the existing problems and potential research directions in the future. In a word, although there are still some problems and the study of acacetin on liver disease is in its infancy, the clinical application prospect of acacetin is anticipated.

## Figures and Tables

**Table 1:** Classification and source of flavonoids.

	Samples	Source	References
Flavonols	Quercetin Kaempferol Fisetin	*Fresh capers* *Anethum graveolens* *Red onion* *Fennel*	[13]
Flavones	Apigenin Acacetin Luteolin	*Chamaemelum nobile* *Tanacetum vulgare* *Origanum vulgare* *Petroselinumcrispum*	[14]
Isoflavones	Daidzein Genistein Glycitein	*Soybeans* *Natto*	[15]
Anthocyanidins	Cyaniding Delphinidin Malvidin	*Apples* *Berries* *Stone fruits* *Grapes*	[16]
Flavanones	Naringenin Hesperetin Eriodictyol	*Orange* *Tangerine* *Lemon* *Grapefruit*	[17]
Flavanols	Epigallocatechin gallate Epicatechin Theflavin-3,3′-digallate	*Cocoa* *Tea* *Berries* *Apricots*	[18]
Chalcones	Lonchocarpin Cardamonin licochalcones	*Lonchocarpussericeus* *Campomanesia adamantium* *Glycyrrhiza urakensis*	[19]

## Data Availability

Data sharing not applicable to this article as no datasheets were generated or analyzed during the current study.

## References

[1] Griffin C., Agbim U., Ramani A., Shankar N., Kanwal F., Asrani S.K. (2021). Underestimation of Cirrhosis-Related Mortality in the Medicare Eligible Population, 1999–2018. Clin. Gastroenterol. Hepatol..

[2] Asrani S.K., Devarbhavi H., Eaton J., Kamath P.S. (2019). Burden of liver diseases in the world. J. Hepatol..

[3] Devarbhavi H., Asrani S.K., Arab J.P., Nartey Y.A., Pose E., Kamath P.S. (2023). Global burden of liver disease: 2023 update. J. Hepatol..

[4] Luk J.M., Wang X., Liu P., Wong K., Chan K., Tong Y., Hui C., Lau G.K., Fan S. (2007). Traditional Chinese herbal medicines for treatment of liver fibrosis and cancer: From laboratory discovery to clinical evaluation. Liver Int..

[5] Peery A.F., Crockett S.D., Murphy C.C., Lund J.L., Dellon E.S., Williams J.L., Jensen E.T., Shaheen N.J., Barritt A.S., Lieber S.R. (2019). Burden and Cost of Gastrointestinal, Liver, and Pancreatic Diseases in the United States: Update 2018. Gastroenterology.

[6] Schlegel A., van Reeven M., Croome K., Parente A., Dolcet A., Widmer J., Meurisse N., De Carlis R., Hessheimer A., Jochmans I. (2021). A multicentre outcome analysis to define global benchmarks for donation after circulatory death liver transplantation. J. Hepatol..

[7] Papich M.G., Davis L.E. (1985). Drugs and the liver. Vet. Clin. N. Am. Small. Anim. Pr..

[8] Almazroo O.A., Miah M.K., Venkataramanan R. (2017). Drug Metabolism in the Liver. Clin. Liver Dis..

[9] Cordell G.A., Colvard M.D. (2012). Natural Products and Traditional Medicine: Turning on a Paradigm. J. Nat. Prod..

[10] Madrigal-Santillán E., Madrigal-Bujaidar E., Álvarez-González I., Sumaya-Martínez M.T., Gutiérrez-Salinas J., Bautista M., Morales-González Á., García-Luna y González-Rubio M., Aguilar-Faisal J.L., Morales-González J.A. (2014). Review of natural products with hepatoprotective effects. World J. Gastroenterol..

[11] Poulsen N.B., Lambert M.N.T., Jeppesen P.B. (2020). The Effect of Plant Derived Bioactive Compounds on Inflammation: A Systematic Review and Meta-Analysis. Mol. Nutr. Food Res..

[12] Shen N., Wang T., Gan Q., Liu S., Wang L., Jin B. (2022). Plant flavonoids: Classification, distribution, biosynthesis, and antioxidant activity. Food Chem..

[13] Kozłowska A., Szostak-Węgierek D. (2022). Targeting Cardiovascular Diseases by Flavonols: An Update. Nutrients.

[14] Hostetler G.L., Ralston R.A., Schwartz S.J. (2017). Flavones: Food Sources, Bioavailability, Metabolism, and Bioactivity. Adv. Nutr..

[15] Danciu C., Avram S., Pavel I.Z., Ghiulai R., Dehelean C.A., Ersilia A., Minda D., Petrescu C., Moaca E.-A., Soica C. (2018). Main Isoflavones Found in Dietary Sources as Natural Anti-inflammatory Agents. Curr. Drug Targets.

[16] Mattioli R., Francioso A., Mosca L., Silva P. (2020). Anthocyanins: A Comprehensive Review of Their Chemical Properties and Health Effects on Cardiovascular and Neurodegenerative Diseases. Molecules.

[17] Chanet A., Milenkovic D., Manach C., Mazur A., Morand C. (2012). Citrus flavanones: What is their role in cardiovascular protection?. J. Agric Food Chem..

[18] Martín M., Ramos S. (2021). Impact of Dietary Flavanols on Microbiota, Immunity and Inflammation in Metabolic Diseases. Nutrients.

[19] Constantinescu T., Lungu C.N. (2021). Anticancer Activity of Natural and Synthetic Chalcones. Int. J. Mol. Sci..

[20] Trefts E., Gannon M., Wasserman D.H. (2017). The liver. Curr. Biol..

[21] Younossi Z.M., Koenig A.B., Abdelatif D., Fazel Y., Henry L., Wymer M. (2016). Global epidemiology of nonalcoholic fatty liver disease-Meta-analytic assessment of prevalence, incidence, and outcomes. Hepatology.

[22] Pouwels S., Sakran N., Graham Y., Leal A., Pintar T., Yang W., Kassir R., Singhal R., Mahawar K., Ramnarain D. (2022). Non-alcoholic fatty liver disease (NAFLD): A review of pathophysiology, clinical management and effects of weight loss. BMC Endocr. Disord..

[23] Paternostro R., Trauner M. (2022). Current treatment of non-alcoholic fatty liver disease. J. Intern. Med..

[24] Vogel A., Meyer T., Sapisochin G., Salem R., Saborowski A. (2022). Hepatocellular carcinoma. Lancet.

[25] Chidambaranathan-Reghupaty S., Fisher P.B., Sarkar D. (2021). Hepatocellular Carcinoma (HCC): Epidemiology, Etiology and Molecular Classification. Adv. Cancer Res..

[26] Galle P.R., Dufour J.-F., Peck-Radosavljevic M., Trojan J., Vogel A. (2020). Systemic Therapy of Advanced Hepatocellular Carcinoma. Futur. Oncol..

[27] Anwanwan D., Singh S.K., Singh S., Saikam V., Singh R. (2019). Challenges in liver cancer and possible treatment approaches. Biochim. Biophys. Acta (BBA) Rev. Cancer.

[28] Khazaei S., Esa N.M., Ramachandran V., Hamid R.A., Pandurangan A.K., Etemad A., Ismail P. (2017). In vitro Antiproliferative and Apoptosis Inducing Effect of Allium Atroviolaceum Bulb Extract on Breast, Cervical, and Liver Cancer Cells. Front. Pharmacol..

[29] Sengupta A., Ghosh S., Bhattacharjee S. (2004). Allium vegetables in cancer prevention: An overview. Asian Pac. J. Cancer Prev..

[30] Ng K.T.P., Guo D.Y., Cheng Q., Geng W., Ling C.C., Li C.X., Liu X.B., Ma Y.Y., Lo C.M., Poon R.T.P. (2012). A Garlic Derivative, S-allylcysteine (SAC), Suppresses Proliferation and Metastasis of Hepatocellular Carcinoma. PLoS ONE.

[31] Hong H., An J.C., de La Cruz J.F., Hwang S.-G. (2017). Cnidium officinale Makino extract induces apoptosis through activation of caspase-3 and p53 in human liver cancer HepG2 cells. Exp. Ther. Med..

[32] Ringelhan M., McKeating J.A., Protzer U. (2017). Viral hepatitis and liver cancer. Philos. Trans. R. Soc. Lond. B Biol. Sci..

[33] Ben Ari Z., Weitzman E., Safran M. (2015). Oncogenic Viruses and Hepatocellular Carcinoma. Clin. Liver Dis..

[34] Odenwald M.A., Paul S. (2022). Viral hepatitis: Past, present, and future. World J. Gastroenterol..

[35] Abutaleb A., Kottilil S. (2020). Hepatitis A: Epidemiology, Natural History, Unusual Clinical Manifestations, and Prevention. Gastroenterol. Clin. N. Am..

[36] Iannacone M., Guidotti L.G. (2021). Immunobiology and pathogenesis of hepatitis B virus infection. Nat. Rev. Immunol..

[37] Jeng W.J., Papatheodoridis G.V., Lok A.S.F. (2023). Hepatitis B. Lancet.

[38] Marcellin P., Gane E., Buti M., Afdhal N., Sievert W., Jacobson I.M., Washington M.K., Germanidis G., Flaherty J.F., Schall R.A. (2012). Regression of cirrhosis during treatment with tenofovir disoproxil fumarate for chronic hepatitis B: A 5-year open-label follow-up study. Lancet.

[39] Liaw Y.-F., Sung J.J.Y., Chow W.C., Farrell G., Lee C.-Z., Yuen H., Tanwandee T., Tao Q.-M., Shue K., Keene O.N. (2004). Lamivudine for Patients with Chronic Hepatitis B and Advanced Liver Disease. N. Engl. J. Med..

[40] Rosen H.R. (2011). Chronic hepatitis C infection. N. Engl. J. Med..

[41] Potthoff A., Manns M.P., Wedemeyer H. (2010). Treatment of HBV/HCV coinfection. Expert Opin. Pharmacother..

[42] González-Grande R., Jiménez-Pérez M., Arjona C.G., Torres J.M. (2016). New approaches in the treatment of hepatitis C. World J. Gastroenterol..

[43] Spearman C.W., Dusheiko G.M., Hellard M., Sonderup M. (2019). Hepatitis C. Lancet.

[44] Crouchet E., Wrensch F., Schuster C., Zeisel M.B., Baumert T.F. (2018). Host-targeting therapies for hepatitis C virus infection: Current developments and future applications. Ther. Adv. Gastroenterol..

[45] Baumert T.F., Berg T., Lim J.K., Nelson D.R. (2019). Status of Direct-Acting Antiviral Therapy for Hepatitis C Virus Infection and Remaining Challenges. Gastroenterology.

[46] Lempp F.A., Ni Y., Urban S. (2016). Hepatitis delta virus: Insights into a peculiar pathogen and novel treatment options. Nat. Rev. Gastroenterol. Hepatol..

[47] Koh C., Heller T., Glenn J.S. (2019). Pathogenesis of and New Therapies for Hepatitis D. Gastroenterology.

[48] Abbas Z., Afzal R. (2013). Life cycle and pathogenesis of hepatitis D virus: A review. World J. Hepatol..

[49] Aslan A.T., Balaban H.Y. (2020). Hepatitis E virus: Epidemiology, diagnosis, clinical manifestations, and treatment. World J. Gastroenterol..

[50] Ma Z., de Man R.A., Kamar N., Pan Q. (2022). Chronic hepatitis E: Advancing research and patient care. J. Hepatol..

[51] Geng Y., Zhang H., Huang W., Harrison T.J., Geng K., Li Z., Wang Y. (2013). Persistent Hepatitis E Virus Genotype 4 Infection in a Child with Acute Lymphoblastic Leukemia. Hepat. Mon..

[52] Todt D., Moeller N., Praditya D., Kinast V., Friesland M., Engelmann M., Verhoye L., Sayed I.M., Behrendt P., Thi V.L.D. (2018). The natural compound silvestrol inhibits hepatitis E virus (HEV) replication in vitro and in vivo. Antivir. Res..

[53] Reuben A., Koch D.G., Lee W.M. (2010). Drug-induced acute liver failure: Results of a U.S. multicenter, prospective study. Hepatology.

[54] Wei G., Bergquist A., Broomé U., Lindgren S., Wallerstedt S., Almer S., Sangfelt P., Danielsson Å., Sandberg-Gertzén H., Lööf L. (2007). Acute liver failure in Sweden: Etiology and outcome. J. Intern. Med..

[55] Reuben A., Tillman H., Fontana R.J., Davern T., McGuire B., Stravitz R.T., Durkalski V., Larson A.M., Liou I., Fix O. (2016). Outcomes in Adults with Acute Liver Failure Between 1998 and 2013: An Observational Cohort Study. Ann. Intern. Med..

[56] Nathwani R.A., Kaplowitz N. (2006). Drug hepatotoxicity. Clin. Liver Dis..

[57] Senior J.R. (2007). Drug Hepatotoxicity from a Regulatory Perspective. Clin. Liver Dis..

[58] Iorga A., Dara L., Kaplowitz N. (2017). Drug-Induced Liver Injury: Cascade of Events Leading to Cell Death, Apoptosis or Necrosis. Int. J. Mol. Sci..

[59] Pu S., Pan Y., Zhang Q., You T., Yue T., Zhang Y., Wang M. (2023). Endoplasmic Reticulum Stress and Mitochondrial Stress in Drug-Induced Liver Injury. Molecules.

[60] Roehlen N., Crouchet E., Baumert T.F. (2020). Liver Fibrosis: Mechanistic Concepts and Therapeutic Perspectives. Cells.

[61] Clichici S., Olteanu D., Nagy A.-L., Oros A., Filip A., Mircea P.A. (2015). Silymarin Inhibits the Progression of Fibrosis in the Early Stages of Liver Injury in CCl_4_-Treated Rats. J. Med. Food.

[62] Tsai J.H., Liu J.Y., Wu T.T., Ho P.C., Huang C.Y., Shyu J.C., Hsieh Y.S., Tsai C.C., Liu Y.C. (2008). Effects of silymarin on the resolution of liver fibrosis induced by carbon tetrachloride in rats. J. Viral Hepat..

[63] Ferenci P. (2016). Silymarin in the treatment of liver diseases: What is the clinical evidence?. Clin. Liver Dis..

[64] Okigawa M., Hatanaka H., Kawano N., Matsunaga I., Tamura Z. (1970). A new glycoside, acacetin-7-glucurono-(1 lead to 2)-glucuronide from the leaves of *Clerodendron trichotomum*. Tetrahedron Lett..

[65] Rendiuk T.D., Krivut B.A., Glyzin V.I. (1978). Spectrophotometric method of determining acacetin in the leaves of the thistle, *Cirsium setosum* (Willd.). Farmatsiia.

[66] Griffiths L.A., Smith G.E. (1972). Metabolism of apigenin and related compounds in the rat. Metabolite formation in vivo and by the intestinal microflora in vitro. Biochem. J..

[67] Singh S., Gupta P., Meena A., Luqman S. (2020). Acacetin, a flavone with diverse therapeutic potential in cancer, inflammation, infections and other metabolic disorders. Food Chem. Toxicol..

[68] Dias M.C., Pinto D.C.G.A., Silva A.M.S. (2021). Plant Flavonoids: Chemical Characteristics and Biological Activity. Molecules.

[69] Terahara N. (2015). Flavonoids in Foods: A Review. Nat. Prod. Commun..

[70] Martens S., Mithöfer A. (2005). Flavones and flavone synthases. Phytochemistry.

[71] Li G.-R., Wang H.-B., Qin G.-W., Jin M.-W., Tang Q., Sun H.-Y., Du X.-L., Deng X.-L., Zhang X.-H., Chen J.-B. (2008). Acacetin, a Natural Flavone, Selectively Inhibits Human Atrial Repolarization Potassium Currents and Prevents Atrial Fibrillation in Dogs. Circulation.

[72] Zhao Y., Cai L., Sui Q., Lin F., Jiang W., Chen J., Lu W., Gao Q. (2016). Facile synthesis of acacetin and its derivatives. Bioorganic Med. Chem. Lett..

[73] Barreca D., Mandalari G., Calderaro A., Smeriglio A., Trombetta D., Felice M.R., Gattuso G. (2020). Citrus Flavones: An Update on Sources, Biological Functions, and Health Promoting Properties. Plants.

[74] Han D.-G., Cha E., Joo J., Hwang J.S., Kim S., Park T., Jeong Y.-S., Maeng H.-J., Kim S.-B., Yoon I.-S. (2021). Investigation of the Factors Responsible for the Poor Oral Bioavailability of Acacetin in Rats: Physicochemical and Biopharmaceutical Aspects. Pharmaceutics.

[75] Yin J., Ma Y., Liang C., Gao J., Wang H., Zhang L.-T. (2019). A Systematic Study of the Metabolites of Dietary Acacetin in Vivo and in Vitro Based on UHPLC-Q-TOF-MS/MS Analysis. J. Agric. Food Chem..

[76] Liu H., Wang Y.-J., Yang L., Zhou M., Jin M.-W., Xiao G.-S., Sun H.-Y., Li G.-R. (2016). Synthesis of a highly water-soluble acacetin prodrug for treating experimental atrial fibrillation in beagle dogs. Sci. Rep..

[77] Pietta P.G. (2000). Flavonoids as antioxidants. J. Nat. Prod..

[78] Berto A., Ribeiro A.B., Sentandreu E., de Souza N.E., Mercadante A.Z., Chisté R.C., Fernandes E. (2015). The seed of the Amazonian fruit *Couepia bracteosa* exhibits higher scavenging capacity against ROS and RNS than its shell and pulp extracts. Food Funct..

[79] Sun L.-C., Zhang H.-B., Gu C.-D., Guo S.-D., Li G., Lian R., Yao Y., Zhang G.-Q. (2017). Protective effect of acacetin on sepsis-induced acute lung injury via its anti-inflammatory and antioxidative activity. Arch. Pharmacal Res..

[80] Wu C., Chen R.L., Wang Y., Wu W.Y., Li G. (2022). Acacetin alleviates myocardial ischaemia/reperfusion injury by inhibiting oxidative stress and apoptosis via the Nrf-2/HO-1 pathway. Pharm. Biol..

[81] Cui Y.-K., Hong Y.-X., Wu W.-Y., Han W.-M., Wu Y., Wu C., Li G.-R., Wang Y. (2022). Acacetin ameliorates cardiac hypertrophy by activating Sirt1/AMPK/PGC-1α pathway. Eur. J. Pharmacol..

[82] Wu D., Wang Y., Zhang H., Du M., Li T. (2017). Acacetin attenuates mice endotoxin-induced acute lung injury via augmentation of heme oxygenase-1 activity. Inflammopharmacology.

[83] Song F., Mao Y.J., Hu Y., Zhao S.S., Wang R., Wu W.Y., Li G.R., Wang Y., Li G. (2022). Acacetin attenuates diabetes-induced cardiomyopathy by inhibiting oxidative stress and energy metabolism via PPAR-alpha/AMPK pathway. Eur. J. Pharmacol..

[84] Liu H., Yang L., Wu H.-J., Chen K.-H., Lin F., Li G., Sun H.-Y., Xiao G.-S., Wang Y., Li G.-R. (2016). Water-soluble acacetin prodrug confers significant cardioprotection against ischemia/reperfusion injury. Sci. Rep..

[85] Pan M.-H., Lai C.-S., Hsu P.-C., Wang Y.-J. (2005). Acacetin Induces Apoptosis in Human Gastric Carcinoma Cells Accompanied by Activation of Caspase Cascades and Production of Reactive Oxygen Species. J. Agric. Food Chem..

[86] Kandhari K., Mishra J.P., Agarwal R., Singh R.P. (2023). Acacetin induces sustained ERK1/2 activation and RIP1-dependent necroptotic death in breast cancer cells. Toxicol. Appl. Pharmacol..

[87] Salimi A., Roudkenar M.H., Sadeghi L., Mohseni A., Seydi E., Pirahmadi N., Pourahmad J. (2016). Selective Anticancer Activity of Acacetin Against Chronic Lymphocytic Leukemia Using Both In Vivo and In Vitro Methods: Key Role of Oxidative Stress and Cancerous Mitochondria. Nutr. Cancer.

[88] Wang S., Lin B., Liu W., Wei G., Li Z., Yu N., Xue X., Ji G. (2020). Acacetin Induces Apoptosis in Human Osteosarcoma Cells by Modulation of ROS/JNK Activation. Drug Des. Dev. Ther..

[89] Alfwuaires M., Elsawy H., Sedky A. (2022). Acacetin Inhibits Cell Proliferation and Induces Apoptosis in Human Hepatocellular Carcinoma Cell Lines. Molecules.

[90] Hsu Y.-L., Kuo P.-L., Lin C.-C. (2003). Acacetin inhibits the proliferation of Hep G2 by blocking cell cycle progression and inducing apoptosis. Biochem. Pharmacol..

[91] Shim H.-Y., Park J.-H., Paik H.-D., Nah S.-Y., Kim D.S., Han Y.S. (2007). Acacetin-induced Apoptosis of Human Breast Cancer MCF-7 Cells Involves Caspase Cascade, Mitochondria-mediated Death Signaling and SAPK/JNK1/2-c-Jun Activation. Mol. Cells.

[92] Hsu Y.-L., Kuo P.-L., Liu C.-F., Lin C.-C. (2004). Acacetin-induced cell cycle arrest and apoptosis in human non-small cell lung cancer A549 cells. Cancer Lett..

[93] Chien S.-T., Lin S.-S., Wang C.-K., Lee Y.-B., Chen K.-S., Fong Y., Shih Y.-W. (2011). Acacetin inhibits the invasion and migration of human non-small cell lung cancer A549 cells by suppressing the p38α MAPK signaling pathway. Mol. Cell. Biochem..

[94] Yun S., Lee Y.-J., Choi J., Kim N.D., Han D.C., Kwon B.-M. (2021). Acacetin Inhibits the Growth of STAT3-Activated DU145 Prostate Cancer Cells by Directly Binding to Signal Transducer and Activator of Transcription 3 (STAT3). Molecules.

[95] Kim H.R., Park C.G., Jung J.Y. (2014). Acacetin (5,7-dihydroxy-4′-methoxyflavone) exhibits in vitro and in vivo anticancer activity through the suppression of NF-kappaB/Akt signaling in prostate cancer cells. Int. J. Mol. Med..

[96] Singh R.P., Agrawal P., Yim D., Agarwal C., Agarwal R. (2005). Acacetin inhibits cell growth and cell cycle progression, and induces apoptosis in human prostate cancer cells: Structure-activity relationship with linarin and linarin acetate. Carcinog..

[97] Cakmakoglu B., Aslan B., Ertugrul B., Iplik E. (2021). Apoptotic effects of acacetin in human colon cancer HT-29 and HCT 116 cells. J. Cancer Res. Ther..

[98] Prasad N., Sharma J.R., Yadav U.C.S. (2020). Induction of growth cessation by acacetin via beta-catenin pathway and apoptosis by apoptosis inducing factor activation in colorectal carcinoma cells. Mol. Biol. Rep..

[99] Yomogida S., Watanabe K., Kanno S.-I., Tomizawa A., Ishikawa M. (2011). Acacetin induces apoptosis in human T cell leukemia Jurkat cells via activation of a caspase cascade. Oncol. Rep..

[100] Lee J.Y., Jun D.Y., Kim K.Y., Ha E.J., Woo M.H., Ko J.Y., Yun Y.H., Oh I.-S., Kim Y.H. (2017). Pharmacologic Inhibition of Autophagy Sensitizes Human Acute Leukemia Jurkat T Cells to Acacetin-Induced Apoptosis. J. Microbiol. Biotechnol..

[101] Zhang G., Dong J., Lu L., Liu Y., Hu D., Wu Y., Zhao A., Xu H. (2023). Acacetin exerts antitumor effects on gastric cancer by targeting EGFR. Front. Pharmacol..

[102] Zhang G., Li Z., Dong J., Zhou W., Zhang Z., Que Z., Zhu X., Xu Y., Cao N., Zhao A. (2022). Acacetin inhibits invasion, migration and TGF-beta1-induced EMT of gastric cancer cells through the PI3K/Akt/Snail pathway. BMC Complement. Med. Ther..

[103] Wang Y., Liu L., Ge M., Cui J., Dong X., Shao Y. (2023). Acacetin attenuates the pancreatic and hepatorenal dysfunction in type 2 diabetic rats induced by high-fat diet combined with streptozotocin. J. Nat. Med..

[104] Liou C.J., Wu S.J., Shen S.C., Chen L.C., Chen Y.L., Huang W.C. (2022). Acacetin Protects against Non-Alcoholic Fatty Liver Disease by Regulating Lipid Accumulation and Inflammation in Mice. Int. J. Mol. Sci..

[105] Ren J., Yue B., Wang H., Zhang B., Luo X., Yu Z., Zhang J., Ren Y., Mani S., Wang Z. (2020). Acacetin Ameliorates Experimental Colitis in Mice via Inhibiting Macrophage Inflammatory Response and Regulating the Composition of Gut Microbiota. Front. Physiol..

[106] Liu J., Wang Y.-G., Yu S.-Y., Li C.-E., Kang S.-M. (2020). Protective effect of acacetin in human periodontal ligament cells *via* regulation of autophagy and inflammation. Pharmazie.

[107] Ha S.K., Moon E., Lee P., Ryu J.H., Oh M.S., Kim S.Y. (2012). Acacetin Attenuates Neuroinflammation via Regulation the Response to LPS Stimuli In Vitro and In Vivo. Neurochem. Res..

[108] Pan M.-H., Lai C.-S., Wang Y.-J., Ho C.-T. (2006). Acacetin suppressed LPS-induced up-expression of iNOS and COX-2 in murine macrophages and TPA-induced tumor promotion in mice. Biochem. Pharmacol..

[109] Wang H., Jiang Z., Pang Z., Zhou T., Gu Y. (2020). Acacetin Alleviates Inflammation and Matrix Degradation in Nucleus Pulposus Cells and Ameliorates Inter-vertebral Disc Degeneration in vivo. Drug Des. Devel. Ther..

[110] Wei Y., Jing J., Peng Z., Liu X., Wang X. (2021). Acacetin ameliorates insulin resistance in obesity mice through regulating Treg/Th17 balance via MiR-23b-3p/NEU1 Axis. BMC Endocr. Disord..

[111] Zhu Y., Bu J., Shi S., Wang H.-Q., Niu X.-S., Zhao Z.-F., Wu W.-D., Zhang X.-L., Ma Z., Zhang Y.-J. (2019). Acacetin protects against cerebral ischemia-reperfusion injury via the NLRP3 signaling pathway. Neural Regen. Res..

[112] Xie M., Wang H., Peng J., Qing D., Zhang X., Guo D., Meng P., Luo Z., Wang X., Peng Q. (2022). Acacetin protects against depression-associated dry eye disease by regulating ubiquitination of NLRP3 through gp78 signal. Front. Pharmacol..

[113] Bu J., Zhang Y., Mahan Y., Shi S., Wu X., Zhang X., Wang Z., Zhou L. (2022). Acacetin improves cognitive function of APP/PS1 Alzheimer’s disease model mice via the NLRP3 inflammasome signaling pathway. Transl. Neurosci..

[114] Hu C.-Q., Chen K., Shi Q., Kilkuskie R.E., Cheng Y.-C., Lee K.-H. (1994). Anti-AIDS Agents, 10. Acacetin-7-O-β-D-galactopyranoside, an Anti-HIV Principle from *Chrysanthemum Morifolium* and a Structure-Activity Correlation with Some Related Flavonoids. J. Nat. Prod..

[115] Critchfield J.W., Butera S.T., Folks T.M. (1996). Inhibition of HIV Activation in Latently Infected Cells by Flavonoid Compounds. AIDS Res. Hum. Retroviruses.

[116] Li J., Zhang K., Bao J., Yang J., Wu C. (2022). Potential mechanism of action of Jing Fang Bai Du San in the treatment of COVID-19 using docking and network pharmacology. Int. J. Med. Sci..

[117] Parvez M.K., Alhowiriny T.A., Al-Dosari M.S., Amina M., Rehman M.T., Al-Yousef H.M., Alanzi A.R., Alajmi M.F. (2023). Inhibition of hepatitis B virus activities by Rhazya stricta-derived acacetin and acetyl-beta-carboline. Exp. Ther. Med..

[118] Xu L., Jiang W., Jia H., Zheng L., Xing J., Liu A., Du G. (2020). Discovery of Multitarget-Directed Ligands Against Influenza A Virus from Compound Yizhihao Through a Predictive System for Compound-Protein Interactions. Front. Cell. Infect. Microbiol..

[119] Cai C., Xu L., Fang J., Dai Z., Wu Q., Liu X., Wang Q., Fang J., Liu A.L., Du G.H. (2021). In Silico Prediction and Bioactivity Evaluation of Chemical Ingredients Against Influenza A Virus from *Isatis Tinctoria* L. Front. Pharmacol..

[120] More G., Lall N., Hussein A., Tshikalange T.E. (2012). Antimicrobial Constituents of *Artemisia Afra* Jacq. ex Willd. against Periodontal Pathogens. Evid. Based Complement. Altern. Med..

[121] Li S., Xu X., Wei L., Wang L., Lv Q. (2022). Acacetin Alleviates *Listeria monocytogenes* Virulence Both In Vitro and In Vivo via the Inhibition of Listeriolysin O. Foodborne Pathog. Dis..

[122] Xie S., Zhang Y., Xu L., Li S., Shen X., Li L., Deng X., Zhou Y. (2022). Acacetin attenuates *Streptococcus suis* virulence by simultaneously targeting suilysin and inflammation. Microb. Pathog..

[123] Kim K.S., Lim D.J., Yang H.J., Choi E.K., Shin M.H., Ahn K.S., Jung S.H., Um J.Y., Jung H.J., Lee J.H. (2013). The multi-targeted effects of Chrysanthemum herb extract against *Escherichia coli* O157:H7. Phytother. Res..

[124] Komape N.P.M., Aderogba M., Bagla V.P., Masoko P., Eloff J.N. (2014). Anti-bacterial and anti-oxidant activities of leaf extracts of *Combretum vendae* (Combretecacea) and the isolation of an anti-bacterial compound. Afr. J. Tradit. Complement. Altern. Med..

[125] Bi C., Dong X., Zhong X., Cai H., Wang D., Wang L. (2016). Acacetin Protects Mice from *Staphylococcus aureus* Bloodstream Infection by Inhibiting the Activity of Sortase A. Molecules.

[126] Li S., Lv Q., Sun X., Tang T., Deng X., Yin Y., Li L. (2020). Acacetin inhibits *Streptococcus pneumoniae* virulence by targeting pneumolysin. J. Pharm. Pharmacol..

[127] Tanase D.M., Gosav E.M., Costea C.F., Ciocoiu M., Lacatusu C.M., Maranduca M.A., Ouatu A., Floria M. (2020). The Intricate Relationship between Type 2 Diabetes Mellitus (T2DM), Insulin Resistance (IR), and Nonalcoholic Fatty Liver Disease (NAFLD). J. Diabetes Res..

[128] Frankowski R., Kobierecki M., Wittczak A., Różycka-Kosmalska M., Pietras T., Sipowicz K., Kosmalski M. (2023). Type 2 Diabetes Mellitus, Non-Alcoholic Fatty Liver Disease, and Metabolic Repercussions: The Vicious Cycle and Its Interplay with Inflammation. Int. J. Mol. Sci..

[129] Wang N., Gao Q., Shi J., Yulan C., Ji W., Sheng X., Zhang R. (2022). Acacetin antagonized lipotoxicity in pancreatic beta-cells via ameliorating oxidative stress and endoplasmic reticulum stress. Mol. Biol. Rep..

[130] Juárez-Reyes K., Brindis F., Medina-Campos O.N., Pedraza-Chaverri J., Bye R., Linares E., Mata R. (2015). Hypoglycemic, antihyperglycemic, and antioxidant effects of the edible plant *Anoda cristata*. J. Ethnopharmacol..

[131] Kwon E.-B., Kang M.-J., Ryu H.W., Lee S., Lee J.-W., Lee M.K., Lee H.-S., Lee S.U., Oh S.-R., Kim M.-O. (2020). Acacetin enhances glucose uptake through insulin-independent GLUT4 translocation in L6 myotubes. Phytomedicine.

[132] Han W.M., Chen X.C., Li G.R., Wang Y. (2020). Acacetin Protects Against High Glucose-Induced Endothelial Cells Injury by Preserving Mitochondrial Function via Activating Sirt1/Sirt3/AMPK Signals. Front. Pharmacol..

[133] Wang X., Xiang J., Huang G., Kang L., Yang G., Wu H., Jiang K., Liang Z., Yang S. (2021). Inhibition of Podocytes DPP4 Activity Is a Potential Mechanism of Lobeliae Chinensis Herba in Treating Diabetic Kidney Disease. Front. Pharmacol..

[134] Wu W.-Y., Li J., Wu Z.-S., Zhang C.-L., Meng X.-L. (2011). STAT3 activation in monocytes accelerates liver cancer progression. BMC Cancer.

[135] He G., Karin M. (2011). NF-kappaB and STAT3—Key players in liver inflammation and cancer. Cell Res..

[136] Yang C., Cai W.-C., Dong Z.-T., Guo J.-W., Zhao Y.-J., Sui C.-J., Yang J.-M. (2018). lncARSR promotes liver cancer stem cells expansion via STAT3 pathway. Gene.

[137] Zhu X., Song G., Zhang S., Chen J., Hu X., Zhu H., Jia X., Li Z., Song W., Chen J. (2022). Asialoglycoprotein Receptor 1 Functions as a Tumor Suppressor in Liver Cancer via Inhibition of STAT3. Cancer Res..

[138] Zeng W., Zhang C., Cheng H., Wu Y.L., Liu J., Chen Z., Huang J.G., Ericksen R.E., Chen L., Zhang H. (2017). Targeting to the non-genomic activity of retinoic acid receptor-gamma by acacetin in hepatocellular carcinoma. Sci. Rep..

[139] Zhang X., Yu C., Zhao S., Wang M., Shang L., Zhou J., Ma Y. (2023). The role of tumor-associated macrophages in hepatocellular carcinoma progression: A narrative review. Cancer Med..

[140] Choi H.-J., Chung T.-W., Ha K.-T. (2016). Luteolin inhibits recruitment of monocytes and migration of Lewis lung carcinoma cells by suppressing chemokine (C–C motif) ligand 2 expression in tumor-associated macrophage. Biochem. Biophys. Res. Commun..

[141] Yue G.G.L., Gao S., Lee J.K.M., Chan Y.Y., Wong E.C.W., Zheng T., Li X.X., Shaw P.C., Simmonds M.S., Lau C.B.S. (2020). A Natural Flavone Tricin from Grains Can Alleviate Tumor Growth and Lung Metastasis in Colorectal Tumor Mice. Molecules.

[142] Eslam M., Sarin S.K., Wong V.W.-S., Fan J.-G., Kawaguchi T., Ahn S.H., Zheng M.-H., Shiha G., Yilmaz Y., Gani R. (2020). The Asian Pacific Association for the Study of the Liver clinical practice guidelines for the diagnosis and management of metabolic associated fatty liver disease. Hepatol. Int..

[143] Loomba R., Friedman S.L., Shulman G.I. (2021). Mechanisms and disease consequences of nonalcoholic fatty liver disease. Cell.

[144] Parola M., Pinzani M. (2024). Liver fibrosis in NAFLD/NASH: From pathophysiology towards diagnostic and therapeutic strategies. Mol. Aspects Med..

[145] Liou C.J., Wu S.J., Chen L.C., Yeh K.W., Chen C.Y., Huang W.C. (2017). Acacetin from Traditionally Used *Saussurea Involucrata* Kar. et Kir. Suppressed Adipogenesis in 3T3-L1 Adipocytes and Attenuated Lipid Accumulation in Obese Mice. Front. Pharmacol..

[146] Jiang Z., Sun H., Miao J., Sheng Q., Xu J., Gao Z., Zhang X., Song Y., Chen K. (2023). The natural flavone acacetin protects against high-fat diet-induced lipid accumulation in the liver via the endo-plasmic reticulum stress/ferroptosis pathway. Biochem. Biophys. Res. Commun..

[147] Zhang Y., Huang Q., Xiong X., Yin T., Chen S., Yuan W., Zeng G., Huang Q. (2023). Acacetin alleviates energy metabolism disorder through promoting white fat browning mediated by AC-cAMP pathway. J. Physiol. Biochem..

[148] Higashi T., Friedman S.L., Hoshida Y. (2017). Hepatic stellate cells as key target in liver fibrosis. Adv. Drug Deliv. Rev..

[149] Li Z.Y., Lv S., Qiao J., Wang S.Q., Ji F., Li D., Yan J., Wei Y., Wu L., Gao C. (2023). Acacetin Alleviates Cardiac Fibrosis via TGF-beta1/Smad and AKT/mTOR Signal Pathways in Spontaneous Hypertensive Rats. Gerontology.

[150] Wei Y., Yuan P., Zhang Q., Fu Y., Hou Y., Gao L., Zheng X., Feng W. (2020). Acacetin improves endothelial dysfunction and aortic fibrosis in insulin-resistant SHR rats by estrogen receptors. Mol. Biol. Rep..

[151] Zhou Y., Wu R., Cai F.-F., Zhou W.-J., Lu Y.-Y., Zhang H., Chen Q.-L., Sun M.-Y., Su S.-B. (2021). Development of a novel anti-liver fibrosis formula with luteolin, licochalcone A, aloe-emodin and acacetin by network pharmacology and transcriptomics analysis. Pharm. Biol..

[152] Li J., Li X., Xu W., Wang S., Hu Z., Zhang Q., Deng X., Wang J., Zhang J., Guo C. (2015). Antifibrotic effects of luteolin on hepatic stellate cells and liver fibrosis by targeting AKT/mTOR/p70S6K and TGF-beta/Smad signalling pathways. Liver Int..

[153] Zheng S., Cao P., Yin Z., Wang X., Chen Y., Yu M., Xu B., Liao C., Duan Y., Zhang S. (2021). Apigenin protects mice against 3,5-diethoxycarbonyl-1,4-dihydrocollidine-induced cholestasis. Food Funct..

[154] Ji J., Yu Q., Dai W., Wu L., Feng J., Zheng Y., Li Y., Guo C. (2021). Apigenin Alleviates Liver Fibrosis by Inhibiting Hepatic Stellate Cell Activation and Autophagy via TGF-beta1/Smad3 and p38/PPARalpha Pathways. PPAR Res..

[155] Tujios S., Stravitz R.T., Lee W.M. (2022). Management of Acute Liver Failure: Update 2022. Semin. Liver Dis..

[156] Stravitz R.T., Lee W.M. (2019). Acute liver failure. Lancet.

[157] Chen T., Li R., Chen P. (2021). Gut Microbiota and Chemical-Induced Acute Liver Injury. Front. Physiol..

[158] Bernal W., Wendon J. (2013). Acute liver failure. N. Engl. J. Med..

[159] Morris S.M., Chauhan A. (2022). The role of platelet mediated thromboinflammation in acute liver injury. Front. Immunol..

[160] Cho H.-I., Park J.-H., Choi H.-S., Kwak J.H., Lee D.-U., Lee S.K., Lee S.-M. (2014). Protective Mechanisms of Acacetin against d-Galactosamine and Lipopolysaccharide-Induced Fulminant Hepatic Failure in Mice. J. Nat. Prod..

[161] Miao J., Yao S., Sun H., Jiang Z., Gao Z., Xu J., Chen K. (2023). Protective Effect of Water-Soluble Acacetin Prodrug on APAP-Induced Acute Liver Injury Is Associated with Upregulation of PPARgamma and Alleviation of ER Stress. Int. J. Mol. Sci..

[162] Jalili C., Akhshi N., Raissi F., Shiravi A., Alvani A., Vaezi G. (2021). Acacetin Alleviates Hepatitis Following Renal Ischemia-Reperfusion in Male Balb/C Mice by Antioxidants Regulation and Inflammatory Markers Suppression. J. Investig. Surg..

[163] Fan L.-H., Li X., Chen D.-Y., Zhang N., Wang Y., Shan Y., Hu Y., Xu R.-A., Jin J., Ge R.-S. (2015). Determination of acacetin in rat plasma by UPLC-MS/MS and its application to a pharmacokinetic study. J. Chromatogr. B.

[164] Jang A.-K., Rashid M., Lee G., Kim D.-Y., Ryu H.W., Oh S.-R., Park J., Lee H., Hong J., Jung B.H. (2022). Metabolites identification for major active components of Agastache rugosa in rat by UPLC-Orbitap-MS: Comparison of the difference between metabolism as a single component and as a component in a multi-component extract. J. Pharm. Biomed. Anal..

[165] Zhang X., Zhang Z.Q., Zhang L.C., Wang K.X., Zhang L.T., Li D.Q. (2021). The development and validation of a sensitive HPLC-MS/MS method for the quantitative and pharmacokinetic study of the seven components of *Buddleja lindleyana Fort*. RSC Adv..

[166] Zhou Y., Tu Y., Zhou Q., Hua A., Geng P., Chen F., Han A., Liu J., Dai D., Wang S. (2020). Evaluation of acacetin inhibition potential against cytochrome P450 in vitro and in vivo. Chem. Interact..

[167] Shokri V., Jalili C., Raissi F., Akhshi N., Ghanbari A. (2019). Evaluating the effects of acacetin versus a low dose of cisplatin drug on male reproductive system and kidney in mice: With emphasis on inflammation process. Andrologia.

[168] Tacke F., Weiskirchen R. (2021). Non-alcoholic fatty liver disease (NAFLD)/non-alcoholic steatohepatitis (NASH)-related liver fibrosis: Mechanisms, treatment and prevention. Ann. Transl. Med..

